# Innovative Sorbents for the Removal of Micropollutants from Water

**DOI:** 10.3390/molecules30071444

**Published:** 2025-03-24

**Authors:** Olga Solcova, Martina Dlaskova, Frantisek Kastanek

**Affiliations:** Institute of Chemical Process Fundamentals ASCR, v.v.i., Rozvojová 135/1, 16500 Prague, Czech Republic; dlaskova@icpf.cas.cz (M.D.); kastanek@icpf.cas.cz (F.K.)

**Keywords:** water-treatment sorbent, sorption, nanotubes, biochar, graphene, metal organic framework

## Abstract

This review summarizes the current knowledge in the field of preparing new and/or innovative materials that can be advantageously used for the sorption of emerging pollutants from water. This paper highlights new innovative materials such as transition metal-modified biochar, zeolites, clays, carbon fibers, graphene, metal organic frameworks, and aerogels. These materials have great potential for the removal of heavy metals from water, particularly due to their large surface area, nanoscale size, and availability of various functionalities; moreover, they can easily be chemically modified and recycled. This paper not only highlights the advantages and ever-improving physicochemical properties of these new types of materials but also critically points out their shortcomings and suggests possible future directions.

## 1. Introduction

The presence of micropollutants in all types of water, including drinking water, poses environmental threats, especially to living organisms and human society. Unfortunately, one of the significant sources of emerging pollutants is municipal wastewater. Current wastewater treatment plants are unable to remove emerging pollutants such as pharmaceuticals [1,2], antibiotics, antibiotic resistance genes, personal care products, hormones, pesticides, and industrial pollutants such as polycyclic hydrocarbons, halogenated substances, per- and polyalkyl-, metallofluorinated, and newly formed nanoplastics, pathogens and many others [3,4,5]. These pollutants can be removed only partially; therefore, they pass through wastewater treatment plants into surface water, and subsequently, they reach drinking water. Municipal wastewater is also a significant source of emerging pollutants.

The results of intensive studies regarding water management issues in recent decades indicate that sorption as a separate capture stage could be a suitable and relatively simple technology for the separation of micropollutants [6]. However, the sorption activity of selected sorbents plays a decisive role within this approach, which is one of the reasons for the search for various innovations in traditional, as well as completely new, innovative sorbents [7].

Currently, there are traditional, simply modified, or activated sorbents [8], either physically, chemically, or by introducing metals, which increase, in particular, the number and amount of surface functional groups and significantly improve the texture of the sorbent. These include classic long-term sorbents such as activated carbon prepared from plant waste, for example, from coconut shells. Increasingly, biochars are being used. They can also be activated or modified in various ways, e.g., by hydrocarbons or magnetized Fe_3_O_4_ [9]. They can be treated either physically, by grinding, steam, CO_2_, etc., or chemically, by acids and bases, e.g., phosphoric acid, sodium hydroxide, etc. Classic sorbents also encompass clays, including their various modifications [10].

Among the innovative sorbents, there are composites of traditional sorbents with metals, preferably replaced by transition metals (Fe, Mn, Mg, V, etc.), which increase the sorption effect, and composites of traditional sorbents with an oxidizing agent, such as hydrogen peroxide, persulfates, etc., producing persistent oxidizing radicals. Innovative sorbents also include metals and organic frameworks, known as MOFs [11]; synthetic zeolites, including the modified ones; aerosols, especially based on silica; graphene; single and multi-walled carbon nanotubes; fullerenes layered double hydroxide sorbents (a class of lamellar compounds formed of positively charged mixed layers of metal hydroxides with an intermediate layer containing charge-compensating anions and water molecules); activated carbon fibers and carbon molecular sieves; simply modified fullerenes and heterofullerenes [12]; and innovative sorbents in nanoparticles, including anchoring nanoparticles (e.g., metals) on biochars or clays.

There is no clear boundary between simply modified and innovative sorbents, especially in relation to composites with other metals or a magnetic component. Innovative sorbents also include nanosorbents because they have a larger surface/volume ratio than, for example, powders or granules, which also require specialized preparation techniques.

## 2. Traditional Modified Sorbents

### 2.1. Activated Carbon

Traditional modified sorbents include activated carbon, which is produced by the high-temperature pyrolysis of selected raw plant materials, especially coconut shells, or from fossil sources of brown coal. In addition to special pyrolysis conditions, the original process of physical and chemical activation is also apparently decisive, with the specific conditions kept secret by producers around the world. However, even with activated carbon, innovative experiments are being conducted, consisting of the activation of pyrolysis products of various parts of plants, such as olive stones, e.g., by applying KOH and H_3_PO_4_. The appearance of bioorganic synthesis will give the process, for example, a solution of lye extract from another plant, e.g., from a banana tree [13], where demineralized lignin shows low reactivity and can be converted at high temperature into microporous activated carbon with the micropore volume of 0.374 cm^3^/g and the high interfacial area of 1305 m^2^/g, at the temperature of 950 °C even in an oxidizing air atmosphere [14]. Pam, 2019 [15] applied an activation solution (choline chloride/urea)/H_3_PO_4_ to plant biomass (palm kernel shell) in an inert atmosphere at 600 °C and achieved a surface area of 1413 m^2^/g and the pore volume of 0.6181 cm^3^/g. Tian et al., 2021 [16] applied coal pyrolysis and activated the product using CO_2_ obtained from the reduction of Fe oxide at 850 °C, thereby obtaining a high microstructure and a high degree of graphitization. They also pyrolyzed plant biomass at the high temperatures of 850 °C in the presence of Fe oxide pellets, which were partially reduced by the carbon product and activated by the evolved CO_2_ to activated carbon with a specific surface area of 320 m^2^/g. Innovative metal oxides (CaO, SrO, MgO) are also used to be applied for modification, e.g., [17] for purification sorption from gases (nitrogen/oxygen separation, biohydrogen purification, etc.). In essence, the world would benefit from innovations in activated carbon production, but commercial activated carbon is currently employed by only a few manufacturers.

### 2.2. Composites with Clays

A number of research studies have already been published and some are continuously investigating the use of natural clay, clay/carbon, and polymer composites, as well as composites with inorganic substances for the removal of organic and inorganic pollutants from water. In addition, to increase the adsorption efficiency of natural clay, the adsorption sites can be improved by chemical and/or thermal activation or incorporation of functional groups into the clay or by modifications of the mineralogical layers of clays, which have a strong affinity for the adsorption of, e.g., emerging pollutants, as well as metal ions. The results of the literature review show that modified clays are better sorbents for the remediation of, e.g., pharmaceutical residues in wastewater than natural clays; moreover, they represent an economically viable and effective option for wastewater treatment; see, e.g., [18]. Clays are relatively powerful sorbents for many organic substances, regrettably with unpredictable results. For example, montmorillonite was able to remove carbamazepine and metformin by more than 70%; however, it does not adsorb, for example, iopamidol [19]. Although iopamidol forms enough –OH functional groups, it is likely that clays might be more suitable for binding with substances containing –NH_2_ functional groups. Clays are often modified with surfactants, such as hexadecyltrimethylammonium bromide (HDTMA) and octadecyltrimethylammonium bromide, which results in a strong hydrophobic bond of around 30 mg/g, which is approximately 10 times greater than unmodified bentonite [20].

Clays modified with these and similar organic substances (octadecyltrimethylammonium (ODTMA), didodecyldimethylammonium-montmorillonite, (DDAB-liposome composite) and benzyldimethylhexadecylammonium (BDMHDA), or polymers such as poly-4-vinylpyridine-co-styrene (HPVP)) for organic micropollutants, e.g., for sorption of simazine and diclofenac, including the preparation of these composites, were reported to by Lelario et al., 2017 [21]. Clays are a truly simple sorbent; their modification is easy. They are effective for various micropollutants and also promising for water-treatment plants in developing countries [22], unfortunately, having been applied only in laboratory settings so far. Furthermore, they are suitable even for waters contaminated with radionuclides and heavy metals, e.g., Cr (VI), especially in anionic form when modified with quaternary ammonium bases, or nanocations Al, Zr, Ti, Fe, and nano-zero valent Fe (NZVI). Kovalchuk I., 2023 [23] described the preparation and mechanism in terms of the sorption capacity of U and Cr up to 300 μmol/g at appropriate pH with the formation of complexes. In principle, the so-called pillared clays can be applied, such as the highly porous material smectite with high hydrophobicity [24]. The most recommended application in the literature is the use of clay modification with cationic surfactants, didodecyldimethylammonium bromide (DDAB), for negatively charged sorbates [25].

Clays are considered natural pollutant absorbers due to their unique properties, such as high specific surface area, surface chemistry, and structural properties. Clay–biochar composites are also commonly applied, usually prepared by pyrolysis of biomass–clay or biochar–clay mixture, e.g., [26]. The composite preparation processes are mostly simple (mixing both components in water, thickening, drying, and pyrolysis); however, the pyrolysis process is crucial for obtaining suitable surface properties of the composite. Regarding the synthesis of clay–biochar composites, montmorillonite, bentonite, kaolinite, and attapulgite clays are widely used. The physicochemical properties of the composite depend on the type of biomass and clay, the ratio of clay to biomass, and the pyrolysis conditions. Pore diameter, pore volume, surface area, and induced functional groups on the sorbent material are the main changes made to the surface of the biochar.

The predominant mechanism of adsorption of organic pollutants onto the composite is realized mainly through electrostatic and hydrophobic interactions, whereas the binding of heavy metal ions and nutrients to the composite occurs through the mechanism of cation exchange and strong covalent bonding [27,28]. Modifications can be achieved by introducing, for example, different functional surface groups or surface electric charges, as well as different hydrophobicities, into one heterogeneous sorbent, which can both increase the sorption capabilities of a specific pollutant and also expand the chances of such a sorbent to be used in multicomponent sorption of organic pollutants and heavy metals in real waters [29,30,31].

There are many positive examples of such a combination. Clay particles and biochar particles can be mixed. However, it is reported that the combination of attapulgite or montmorillonite/biochar sorbs 17-estradiol and ciproflaxin is better than heterogeneous mixtures of biochar and clay alone [32].

### 2.3. Biochar Modified with Transition Metals

It appears that the biochars prepared by pyrolysis of biomass modified with transition metals at high temperatures up to around 700 °C have the presence of resistant oxidation radicals, which is manifested by the fact that the sorbed organic substances are at the same time also somewhat oxidized. This is a remarkable phenomenon that can be appreciated, for instance, with antibiotics, when adsorbed antibiotics are removed, for example, from water; however, those microorganisms that have become resistant can still emit antibiotic-resistant genes into the environment. By oxidizing them with the redox activity induced, for example, by the presence of Mn oxide, this phenomenon disappears, which is fundamentally positive. The oxidative activity of such modified biochar reveals if the metal oxide, or Mn(II), is added to the biomass before the pyrolysis. It has been confirmed that pyrolysis itself can generate persistent radicals in biochar [33], while the presence of transition metals enhances the process.

The synergistic effect of the surface phenomena of biochars and MnO_x_ will allow for a more powerful generation of oxidative radicals and sorption/degradation of, for example, antibiotics, as shown, and the oxidation mechanism explained using the example of cephalexin, for example, by [34]. The modification is remarkably easy: the sorted biomass below 100 mesh is mixed with an aqueous solution of MnCl_2_, dried at 80 °C, and subsequently pyrolyzed. Related to the oxidative radicals •OH generated by the activation of dissolved oxygen in water and the contribution of the phenolic functional group -OH, the oxidation reaction is drastically enhanced by the presence of MnO_x_. The application of magnetic nanomaterials (e.g., Fe_2_O_3_ and Fe_3_O_4_) to biochar is a state-of-the-art technique for the sustainable and safe use of biochar within the removal of contaminants while simultaneously increasing its sorption efficiency.

Spinel ferrites (MFe_2_O_4_, M = Mn, Co, Ni, Mg, Zn), typical magnetic metal oxides, are currently used in various environmental applications, especially in sorption [35]. Their unique physicochemical properties (i.e., large surface area, high stability, and strong magnetism) allow ferrites to be the potential sorbents of contaminants present in water [36]. Recently, biochar-ferrite composites have received increasing attention. In such cases where ferrites are integrated with biochar, the composites exhibit excellent magnetic properties, leading to excellent sorption properties. They can also be applied to the removal of metal ions, e.g., Sb(III), an order of magnitude more toxic than Sb(V), oxidized to Sb(V) by such spinel on biochar and adsorbed on the biochar [37]. Magnetic biochar not only facilitates separation but also exhibits better physicochemical properties and adsorption performance towards emerging pollutants [38]. There are several methods for magnetic biochar preparation. Impregnation-pyrolysis and coprecipitation methods, which account for approximately 69.6% of all preparation techniques, are the most commonly used ones [39].

## 3. New or Innovated Types of Sorbents

Various solid substances—sorbents in nano-sized particles, advantageously using their large surface area/mass ratio—can also be considered a new type of adsorbent. However, their preparation and subsequent separation from water appears rather expensive. It is also necessary to consider the toxicity of nanoparticles in general.

### 3.1. Carbon-Based Sorbents

Carbon nanostructures are widely used as adsorbents due to their excellent surface properties, adaptability, tunable structural changes, and high chemical stability. Various reviews provide information on the latest research in the production and use of carbon-based nano-adsorbents with an emphasis on carbon nanotubes, graphene, and graphene-derived adsorbents. Their use in the extraction of chemicals, pharmaceuticals, and hormone-disrupting pollutants has been and still is being investigated, including the classification of these pollutants, their processing, and new perspectives for increasing the use of carbon nano-adsorbents.

In particular, these are carbon-based sorbents, such as carbon nanotubes, graphene, graphene oxide, and fullerene. Related to the textural properties, special bonds with a sorbate during the sorption should be applied. These include, for example, π–π non-covalent interactions. In general, π–cation interactions are attributed to the electrostatic interaction among the aromatic ring of the basic carbon material, such as carbon foams, carbon nanotubes, activated carbon, graphene, the metal cations (it is a kind of electrostatic interaction close to van der Waals forces) and biochars, too. A well-prepared biochar contains a graphitic structure with π-electrons and allows the adsorption of metal cations. π–π non-covalent interactions, also known as π–π donor-acceptor interactions, between the π-electron in the carbon sorbent and the π-electron between the aromatic ring of the adsorbate. These are important bonding interactions, in addition to the hydrophobic interactions of the carbon sorbent-sorbate, e.g., biochar-hormone. Biochars such as activated carbon are tested to be made microporous to sorb molecules with dimensions smaller than micropores. However, activated carbon is still considered a supersorbent, e.g., [40]. It should be noted that sorbents with a graphitic structure can successfully sorb both organic hydrophobic substances and heavy metals, while the degree of sorption power in mixed contamination of organic and inorganic pollutants cannot be predicted for real contaminated water. It depends on pH, temperature, composition, and concentrations of pollutants. Such an experiment is significantly decisive.

#### 3.1.1. Carbon and Metal-Based Nanotubes

Carbon nanotubes (CNTs) have a relatively good interfacial surface and a dense network of adsorption sites. They are naturally hydrophobic and can be produced by an electric discharge process (arc discharge) between graphite electrodes, 50–100 A, 20 volts between electrodes, in an inert environment of argon or helium and at the pressure of 500 Torr [41], chemical vapor deposition (CO and gaseous hydrocarbons at high temperatures up to 1100 °C, deposited on Co or Ni and MgO supports) or laser ablation. They are particularly useful for hydrophobic substances and especially aromatic pollutants due to π–π interactions and van der Waals forces. Their functional groups can also be advantageously modified, e.g., by introducing -COOH and -NH. They can be used as adsorbents for a number of pollutants [42,43], especially for organic chemicals, heavy metals, and radionuclides [44].

**Figure 1:**
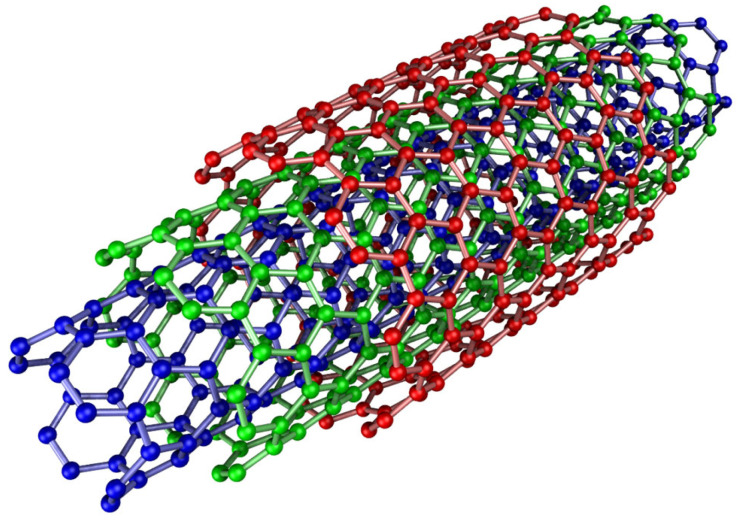
Multi-walled nanotube [45].

However, most of the functional applications of CNTs are limited to laboratory-scale experiments. The feasibility of their large/industrial applications with cost-effective synthesis routes and assessment of their toxicity with better simulation of adsorption mechanisms need to be further studied. Oxidation modification using oxidizing solutions will increase their interfacial area up to values above 100 m^2^/g [41] (Figure 1).

Various techniques have been developed for the production of carbon nanotubes [45], including arc discharge, laser ablation, high-pressure carbon monoxide disproportionation, and chemical vapor deposition. Most of these processes are carried out in a vacuum or with process gases; see [46]. For experiments purposes, carbon nanotubes, CNTs, are usually obtained from manufacturers. For example, multi-walled nanotubes are supplied as already functionalized, e.g., with -OH groups (up to 2.60% -OH groups) and -COOH groups (up to 1.63%) from Nanostructure & Amorphous Materials, Inc. (Kety, TX, USA), or functionalized with oxygen groups from other manufacturers, e.g., (Nanomaterials and Graphene Technology Center (CTNano)/UFMG, Belo Horizonte, Brazil). However, there are many other manufacturers, e.g., Chinese Timesano [47]. The nanotubes are usually further impregnated, e.g., with an extract from various green plants with Fe (II) added (the authors call it a green synthesis [48]), and then applied to the sorption of various pharmaceuticals or bisphenol A in water, so far, only in laboratory size in mL units. An optimal choice of variously functionalized nanotubes obviously depends on the chemistry of the emerging pollutant. Moreover, nanotubes can even be applied as photocatalysts for the removal of pharmaceuticals and personal care products, including cosmetics, in water [49].

The mechanisms of adsorption of emerging pollutants, including antibiotics, into the surface of CNTs are mainly associated with chemisorption and physisorption. Physical adsorption was based on hydrogen bonding and pore filling. On the other hand, chemical adsorption was realized by electrostatic interaction and hydrophobic reactions. CNTs are more mesoporous than activated carbon, which makes them more favorable for the adsorption of larger (bulk) organic compounds. Due to adsorption, CNTs can remove pollutants such as bisphenol A, estriol, 17 α-ethinylestradiol, and pharmaceuticals such as diclofenac, sulfamethoxazole, and amoxicillin, making them potentially valuable adsorbent for an industrial use connected with wastewater treatment. Research in CNTs primarily reports on their use related to membrane filtration. During photocatalytic degradation, variants of TiO_2_/CNT photocatalysts were identified and demonstrated increased activity of solar light, increased adsorption properties, and reduced energy requirements in relation to UV light.

Titanate nanotubes, for example, exhibit an exceptional sorption capacity connected with their texture and ion exchange capacity, making them suitable candidates for the sorption of organic pollutants. The surface of the tubes is either positively charged in acidic solutions or negatively charged in alkaline ones. When modified with surfactants and/or other organic cations, they acquire a positive surface charge in acidic and alkaline solutions and can sorb anions well. TiO_2_ nanoparticles are mixed with NaOH solution, heated in an autoclave at 150 °C, and after cooling and deionization with HCl solution, they form a typical nanotube structure [50].

Nevertheless, there is a problem with the high costs related to the production of CNTs for their applications in water and wastewater treatment plants, as they are, presently, only tested on a laboratory scale; therefore, other ways are being sought to produce them on a large scale economically; see [43].

Simultaneously, there is uncertainty regarding human toxicity and the associated dosing of CNTs into contaminated water. A standardization of toxicity testing and the development of a reliable risk assessment protocol is essential to better assess the potential risks to human health and the environment associated with exposure to both CNTs and nanoparticles in general.

#### 3.1.2. Graphene/Graphene Oxide

Graphene, also called a stellar universal material, with its exceptionally high electrical conductivity, electron mobility, thermal conductivity, thermal stability, optical transparency, and mechanical strength, has attracted great attention from scientists and engineers in the fields of materials, chemistry, physics, energy, and environment in the last decade. It is prepared by, for example, direct exfoliation of graphite, chemical vapor deposition of hydrocarbons, laser-induced direct synthesis of graphene, laser-etched graphene oxide in the dry state, unzipping of carbon nanotubes, polycondensation of polycyclic hydrocarbons, etc., [51,52]. Graphene evinces unparalleled multifunctional properties and enables versatile applications in novel conductive additives, reinforcements, and fillers, such as separation membranes, sensors, anti-corrosion coating, catalysts, electromagnetic shielding, lubricants, as flexible electrode materials in electrochemical and electronic devices, including photovoltaic cells, supercapacitors, rechargeable batteries, as sensors, transistors, light-emitting diodes, adsorbents and absorbers, catalysts, electro-optic modulators, terahertz emitters and detectors and semiconductors, and has been used in the production of photovoltaic cells [53,54]. Especially in terms of high performance and cost-effectiveness, graphene is expected to even surpass expensive carbon nanotubes [55,56].

**Figure 2:**
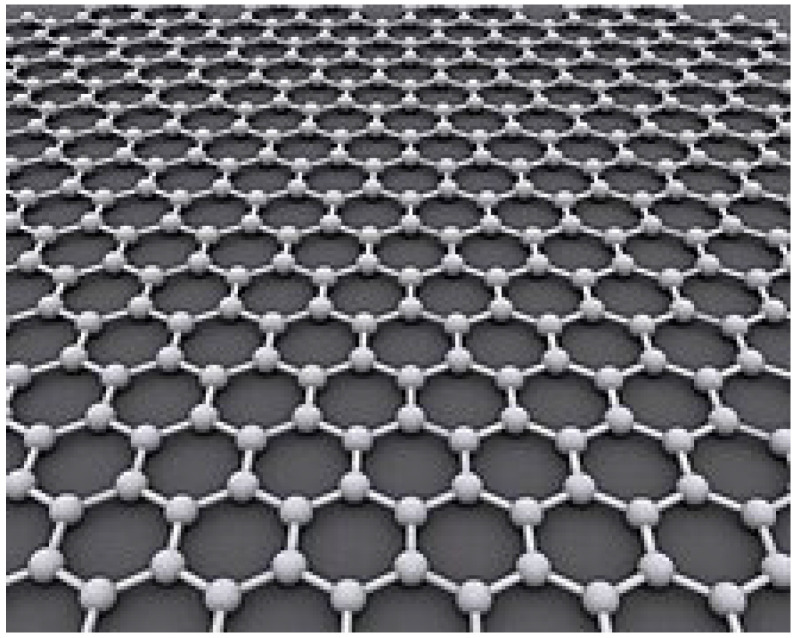
Structure of graphene [57].

Graphene is a super thin form of carbon with a graphite-like structure. It is one of the strongest materials known in the world although being just one atom thick [57] (Figure 2).

**Figure 3:**
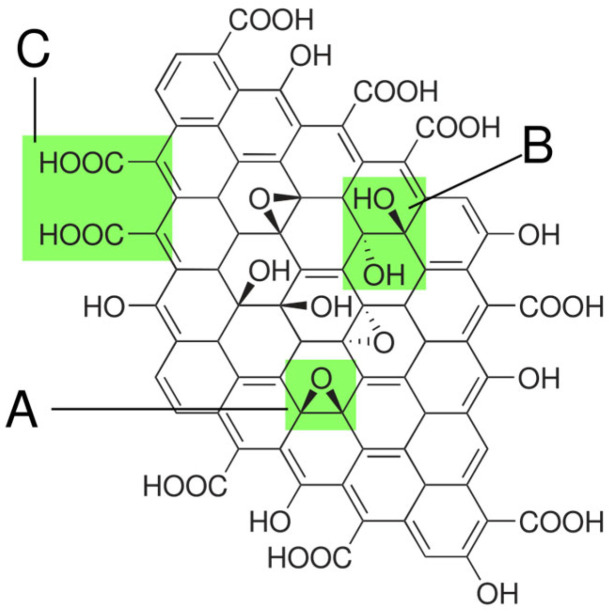
The structure of graphene oxide: A—epoxy-bridges, B—hydroxyl groups, C—carbonyl groups; [60].

Graphene oxide (GO) is an oxidized form of graphene. It is a carbon-based nanomaterial obtained by chemical oxidation of natural graphite or carbon nanofibers with strong oxidants. This material had been known much earlier than the original graphene and is considered one of the most important material precursors of graphene. Graphene and GO appear to be ideal sorbents for micropollutants [58] due to their huge surface area (e.g., the theoretical value for graphene is 2630 m^2^ g^−1^) and the hexagonal arrangement of carbon atoms in the graphene sheets, which are ideal for strong interactions with other molecules. However, graphene is insoluble and difficult to disperse in all solvents, including water. In contrast, GO has a large number of oxygen atoms on the GO surface in the form of epoxy, hydroxyl, and carboxyl groups; therefore, GO is much more hydrophilic than graphene and can form stable colloidal suspensions. GO provides rich functional groups for hydrogen bonding and electrostatic interactions with organic compounds or metal ions. Due to the presence of oxygen functional groups, GO must be reduced by thermal or chemical processes to obtain graphene. However, these procedures do not restore the graphene structure completely, and some O-groups remain bound in the basal plane and modify the properties of graphene oxide. This new material is referred to as a reduced graphene oxide (rGO) (Figure 3). The synthesis of graphene oxide is specifically carried out by the oxidation of natural graphite flakes, e.g., according to [59].

According to the literature [60,61,62], graphene oxide (GO) is characterized by excellent hydrophilicity, high surface area, a large number of functional groups, and strong π interactions, which allows its use in thin films for the removal of emerging pollutants. The graphene lattice contains various functional groups, which, together with hydrophilicity and π–π bonds, participate in the sorption of various pollutants. It is impossible to predict which characteristics will particularly prevail, as they depend on the subtle nuances of the production, as well as the properties of different graphene oxides and the composition of real solutions. Changes in the surface charge at different pH values can be detected, for example, by measuring the zeta potential. The evolution of oxygen-containing functional groups bound to rGO was analyzed by Fourier transform infrared spectroscopy and ultraviolet absorption spectroscopy. The results showed that the edge phenolic hydroxyl and carboxyl groups contribute more to the negative surface charge than the hydroxyl and epoxy groups in the basal plane [63]. Recently, graphene, graphene oxide, and rGO have been tested for the removal of many pollutants, such as heavy metals, dyes, phenols, metals, organic and inorganic pollutants, etc. Single-layer graphene is hydrophilic, whereas multilayer graphene exhibits hydrophobic properties. The hydrophobicity of graphene can be influenced by the number of layers and the presence of functional groups on its surface. This property makes graphene versatile for applications that require specific water interaction properties [64].

The large-scale commercial use of graphene is currently limited by the lack of economically viable processes associated with the preparation of graphene nanomaterials of large linear dimensions [65].

Graphene and graphene oxide are considered excellent sorbents in the literature [66,67]. Graphene-based materials show excellent adsorption of heavy metal ions, dyes, oils, herbicides, pesticides, bisphenol A, carbamazepine, and other generally emerging pollutants [68,69].

It is mainly graphene oxide in the form of magnetic particles that have recently been used as a sorbent for wastewater treatment in applications such as the separation of heavy metals (mercury, cadmium, copper, chromium, arsenic) or organic substances (antibiotics, dyes, e.g., reactive black 5, etc.). The review [70] presents selected examples—mostly from the literature, examining, among other things, the method of synthesis (impregnation, coprecipitation), adsorption kinetics, thermodynamics, isothermal studies, and applications, in comparison with other adsorbents. Currently, there are a number of suppliers of these materials, e.g., the company Nanografi Nano Technology, Ankara, Turkey, which has been producing and supplying advanced nano- and micromaterials, carbon materials, graphene, graphene oxide (GO), reduced graphene oxide (rGO), and carbon nanotubes (CNT) since its establishment in 2011 [71].

However, it is not easy to clearly assess whether graphene or graphene oxide is a better adsorbent. Experiments with real wastewater might probably provide an answer; however, so far, no similar comparison exists. It would be particularly interesting to see how micropollutants, PFAS, antibiotics, and possibly antibiotic-resistant genes are sorbed: Some experiments have already been successfully carried out, e.g., with genes [72,73,74]; nevertheless, the results are still problematic. Moreover, the synthesized graphene-based photocatalysts successfully removed ampC and significantly reduced ecfX abundance of Pseudomonas aeruginosa, but sul1, ermB, and 23S rRNA for enterococci sequences were found to be persistent throughout the treatment with all catalyst types. This shows, among other things, existing reserves in this direction, where even innovative sorbents are not yet ready to completely remove biological pollutants, and further research is necessary in this direction. Correspondingly, the question arises whether a similar problem could still be dealt with more easily by a somewhat simpler, albeit also innovative, sorbent, e.g., diethylaminoethyl cellulose (a positively charged resin used in ion exchange chromatography, a type of column chromatography, for the separation and purification of proteins and nucleic acids) [75].

Effective and inexpensive sorbents are necessary for the removal of perfluoroalkyl and polyfluoroalkyl substances (PFAS) from water sources. Carbon-based materials are promising adsorbents for PFAS. In this work, the potential of graphite oxide (GO) and its derivatives as PFAS adsorbents was investigated by studying the adsorption of perfluorooctanoic acid (PFOA), a model PFAS molecule [76]. PFAS removal by means of sorption is considered a kind of test of the suitability of a sorbent for the removal of serious pollutants from water, e.g., [66]. Graphene oxide has been shown to have such an ability. However, in some cases, it was primarily the modification of graphene oxide-cyclodextrin which was effective; see, e.g., [77]. Graphene oxide seems to be considered a material that possesses the properties of quality sorbents. Considering the innovative carbon materials for real-world applications, it seems most promising to produce them in necessary quantities at a reasonable price, e.g., for tertiary stages of wastewater treatment plants.

#### 3.1.3. Fullerenes

**Figure 4:**
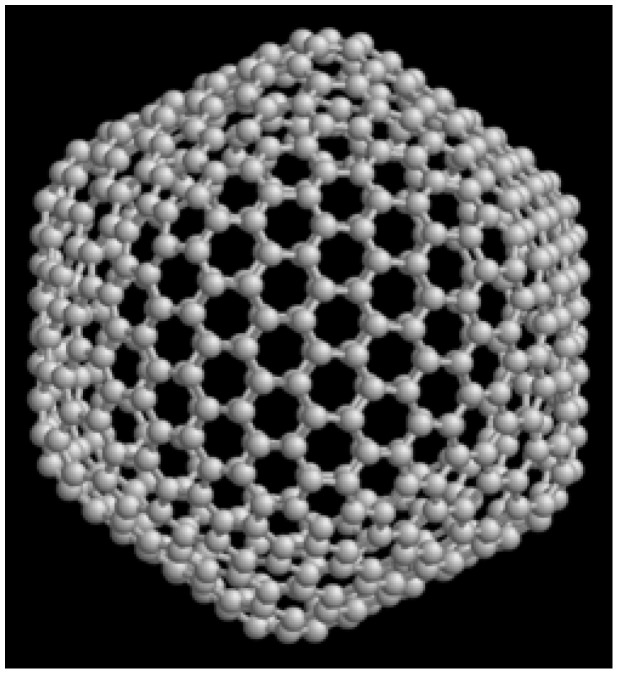
The structure of fullerene C_540_ [79].

Fullerenes are molecules formed by carbon atoms arranged in a layer of pentagons and hexagons with atoms at the vertices, which are spatially rolled into a closed shape (most often into the shape of a sphere or ellipsoid). Owing to this structure, they are extremely resistant to external physical influences. Another atom, several atoms, or a small molecule can be enclosed in the cavity of a fullerene molecule. The most stable fullerene known so far contains 60 carbon atoms. Fullerenes are most often artificially prepared in an electric arc with carbon electrodes or by pyrolysis of organic compounds in a controlled flame. Due to their properties, they belong to the carbon innovative sorbents, e.g., [78,79] (Figure 4).

A feasible ecological magnetic synthesis of fullerenes was realized from waste PET bottles [80]. A mixture of crushed raw material and ferrocene was heat-treated at the high temperature of 800 °C (Ferrocene is an organometallic compound consisting of two cyclopentadienide aromatic rings, between which divalent iron is coordinated by a pentahaptic bond; commercially available). In the case where both cationic and anionic synthetic dyes were sorbed, the sorption mechanism was realized mainly through electrostatic interaction, π–π interaction, and hydrogen bonding. New fullerene composites were synthesized, e.g., fullerene containing nitrogen in the C_24_N_24_ type molecule, doped, e.g., with Ga for dye adsorption [81].

Advances in the field of fullerenes have been observed mainly in the form of nanocomposites. The use of fullerene nanocomposites has also been found in the field of membranes. The addition of fullerene nanoparticles changes the microstructure and physical properties of nanocomposite membranes. Generally, changes in the structure of fullerene nanocomposites in the membrane led to a greater separation of salts, desired metals, toxic metal ions, microorganisms, etc. [82]. Although fullerenes have great potential for adsorption from water, their cost is too high, which unfortunately limits their use [83].

### 3.2. MOF—Metal Organic Framework

**Figure 5:**
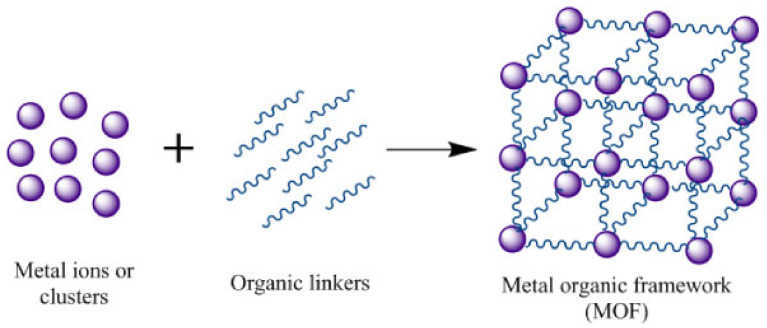
MOF [86].

MOFs are highly ordered porous organic-inorganic hybrid materials formed by the coordination of metal ions with organic ligands, commonly known as absorbent coordination polymers. They are crystalline materials formed from metal ions or clusters bridged by organic ligands to form one-, two- or three-dimensional infinite networks. Metal organic frameworks (MOFs) are formed by metal ions/clusters coordinated by organic linkers (or bridging ligands). There is a variety of synthetic methods (solvothermal/hydrothermal, microwave-assisted, electrochemical, mechanochemical, sonochemical) and linkers, and over 90,000 different MOFs can be constructed. MOF ligands have a rigid backbone with various functional groups, including mono-, bi-, tri-, or tetracarboxylic acids (e.g., 2,2′-Bipyridine-5,5′-dicarboxylic acid, Benzene-1,3,5-triyltriboronic acid, etc.), hydroxyl groups, nitrogen-containing heterocyclic structures or mixed functional groups. This diversity enables a wide range of applications, e.g., [84]. Each MOF ligand carries two sets of functional groups. One set is binding to metals and forms the MOF structure. The other set is involved in secondary reactivity after the MOF is formed, which opens up endless possibilities in the functionality of the material. The physical and chemical properties of the connectors play a crucial role in the properties of the resulting MOF, e.g., [85,86] (Figure 5).

A characteristic feature of MOFs is their permanent porosity. These clusters are often formed in situ, while linkers are usually preformed. The geometry and connectivity of the linker determine the structure of the resulting MOF. By adjusting the geometry, length, ratio, and functional group of the linker, the size, shape, and internal surface properties of the MOF can be tuned for a targeted application. The issue of MOFs forms a separate special chapter of metal organic chemistry, which became of interest mainly in the last decade, e.g., [87]. In a critical review by [84], the advances in MOF synthesis with a focus on the linker design are shown.

MOF structures can be designed and synthesized according to specific functions due to the rich geometry of metal nodes and organic ligands of MOFs and diverse connection modes [84]. Furthermore, it is the diversity of MOFs that has led to a wide range of applications in several areas, including the catalysis and sorption of substances from water and air. Different research teams have created different types of MOFs; see, e.g., the review by [88]. In addition to the sorption properties, their stability is important, and they are influenced by the conditions of their synthesis (temperature, pH, etc.). Highly valent metal units, including Zr^4+^, Fe^3+^, and Cr^3+^, are usually chosen for combinations with carboxylate bridges to form compounds stable in water [89,90]. Covalently bonded organometallic frameworks with special attention paid to MOF nanocomposites, therefore having a number of characteristic properties, including improved porosity, functionalized morphology, and high surface area, are often homogeneously mixed with magnetically active nanoparticles to produce magnetic nanocomposites for catalyzing a wide range of applications, including slow-release fertilizers/drugs, catalysis and, in our interest, especially adsorption. MOFs can be “tailored” to different pollutants, with POE being considered the best choice in terms of sorption capabilities. Regarding the applications of MOFs in the removal of pollutants from water, it is possible to synthesize MOFs particularly targeted at a specific and highly efficient sorption of particular pollutants. They can be tuned for preferential hydrophobicity, electrostatic interactions, π–π exchange, hydrogen H- bonding, bonds to acid H^+^ or base OH^−^ groups, and a number of such MOFs can be synthesized; for example, zirconium-based MOFs for polycyclic aromatic hydrocarbons, where the available MOFs have their characteristic designations (UiO-66 or MOF-5 for ciproflaxin, etc.). Some MOFs also function as photocatalysts, e.g., magnetically removable composite with carbide nitride C_3_N_4_@CoFe_2_O_4_/Fe_2_O_3_. Furthermore, MOF composites are suitable as catalysts for the Fenton process and MOFs with enzymes for biodegradation. MOF with herbicide hydrolase (QpeH@ZIF-10) removes herbicides from water. They also function as photocatalysts (LED lamp 5W) for the decomposition of synthetic dyes. The MOF synthesis—in this case, MOF/CuWO4—is easy [91]. Enzymes can also be immobilized into MOF. The most practical way of immobilizing enzymes is surface adsorption, which can easily utilize a large number of enzymes. It relies mainly on weak interactions, such as hydrogen bonds, π–π interactions, and molecular interactions between the enzyme and MOFs. It is also possible to incorporate microorganisms into the MOF. MOFs provide them with protection from toxic effects common in wastewater, and, related to that, microorganisms can, for example, sorb heavy metals effectively [92,93].

The current literature generally describes MOFs as highly effective sorbents for pollutants in both water and gases, e.g., as a hydrogen reservoir [94,95].

The use of water-stable MOFs, such as MOF-235, MIL-53, MIL-100, MIL-101, UiO-66, ZIF-67 and ZIF-8; their adsorption efficiency; and functionalized MOFs; magnetic MOFs, MOF-polymers; graphene oxide-MOF composites; and CNT-MOFs, in comparison with the commercial sorbents such as zeolites and activated carbons and their use for the removal of heavy metals, dyes, pharmaceuticals, personal care products, pesticides and herbicides from polluted water sources, are also mentioned.

However, regarding mixtures, which are typically associated with contaminated waters, it is essentially impossible to select the best combination of one specific MOF a priori. However, this offers the possibility of “infinite” tests for selecting the most suitable MOF. Materials for environmental remediation, including MOFs, must not only meet the basic requirements of low cost, high efficiency, and stability but also can remove multiple contaminants simultaneously. However, when several types of contaminants coexist, it is difficult to explain the complex mechanism. Only a few relevant reports deal with the mechanism of removing multiple, simultaneously existing contaminants. For example, [95] tried to find it. Among others, he cites the work [96], which combined the poly(arylene sulfide/sulfone) polymer with MOF (here UiO-66) by the electrospinning technology using a nanofibrous membrane, which had been supposed to absorb some organic pollutants and metals together. Here, UiO-66 (Universitetet i Oslo) is a metal organic framework made up of [Zr_6_O_4_(OH)_4_] clusters with a 1,4-benzodicarboxylic acid framework (struts), often applied in laboratory experiments.

A great advantage of MOFs is their good sorption of antibiotics from water, which, presently, regarding the quality of water, represents a broad issue, as over 250 antibiotics can be found in water today [97,98]. Moreover, MOFs are primarily associated with their excellent ability to sorb antibiotic-resistant genes, which provides a huge potential future perspective [75,96].

The real application of MOFs regarding wastewater is far from easy. Considering the working conditions of MOFs, wastewater from rivers and wastewater treatment plants (WWTPs) involves various complex interactions among substances, and the variables simulated in the laboratory (such as pH, temperature, and pollutant concentration) are far from representing the real environment. In real water, there are also other active molecules, such as pollutant particles and metal salts, which can cause competitive adsorption on the binding sites of MOFs. Most of the experiments have not been performed in real water samples, which is one of the problems that MOFs face in practical applications. The versatility of various MOFs is astonishing; nevertheless, it appears essential to make research efforts to find and specify the efficient and universally effective individual and complex combinations of MOFs to work as sorbents ideally and, what is more, their economically relevant production on mass scales needs to be solved too, as it is also still unavailable. Currently, regarding the unexpected military conflicts in the world, there appear to be a number of promising applications related particularly to the removal of antibiotics and explosives. Such applications involve MOFs [89,99], where Zr-MOF, specifically Zr-MOF UiO-66-NH2, could be synthesized for the specific purposes, e.g., [100].

There are a significant number of similar reports focusing on how different synthesized MOFs sorb under various specific conditions. Review publications, such as the one by Liu et al., 2023 [88], are now available; however, there are still some further remaining challenges: (1) improving the regenerability/regeneration of MOFs; and (2) better understanding the interaction of contaminants with MOFs.

For thousands of different MOF individuals, it is essentially impossible to experimentally test all variations concerning how they sorb various micropollutants from prepared waters, nor are the results of sorption of micropollutants from real wastewater known. It seems that the sorption efficiency is high; therefore, it would be appropriate to experimentally test how MOFs would behave in real waters containing very low concentrations of micropollutants in the order of micro- and nanograms/l. It is known that MOFs sorb fluorides particularly well from water [101]. It appears that MOFs are essentially universal sorbents, as they can sorb both organic emerging pollutants and metals. It also seems possible that they would adsorb, for example, rare metals from, e.g., thermal waters, which would certainly be worth testing. It is difficult to predict what the future holds for MOFs today; nevertheless, it is evident they deserve separate detailed research, which is beyond the scope of this minireview. Some experiments already exist. The current paper [102], which confirms the universality of the sorption activity of MOFs, is highly beneficial.

In 1999, Li and his co-workers [103] synthesized MOF-5 using zinc nitrate, 1,4-BDC, chlorobenzene, and DMF, which has the formula Zn_4_O(BDC)_3_-(DMF)_8_-(C_6_H_5_C_l_). It was found [102] that MOF-5 is more effective than ZnO and TiO_2_ for the photocatalytic degradation of waste organic contaminants due to its ability to absorb visible light and exhibit a broad absorption spectrum in the visible region. Lal et al., 2024 [102] report MOFs with various metals (Ni, Co, Cu, Zn, Ti, etc.) as photocatalysts. It is, therefore, remarkable that some MOFs can function as photocatalysts, which could perhaps be used in innovative tertiary stages of water-treatment plants.

It would certainly be beneficial to verify some MOFs in real waters to evaluate the possibilities of producing such MOFs on a large scale and whether it could be done cost-effectively. However, it seems that, despite their versatility, MOFs are connected with particular major disadvantages for industrial applications. For example, MOFs usually have a low sorption capacity. Properly speaking, they can absorb only a limited amount of a certain contaminant before they become saturated [95]. This limits their effectiveness for large-scale applications. Moreover, MOFs are usually more expensive than other types of sorbents, such as activated carbon and zeolites, which makes them less attractive for commercial applications. MOFs are also prone to deactivation, i.e., they can lose their adsorption properties over time. This can lead to a decrease in efficiency and an increase in costs. MOFs are also difficult to manufacture due to their complexity and the need for precise control of synthesis conditions.

Apparently, the future of MOFs is to use them to create systems that would be more selective and effective in removing specific types of contaminants. In addition, MOFs could also be used to create systems tailored to specific applications, such as removing specific types of pollutants or heavy metals. Overall, MOFs have enormous potential for water remediation applications and could revolutionize the way water with special compositions is cleaned and treated, rather than in a wastewater treatment plant. In essence, this conclusion could also apply to the other innovative sorbents mentioned here.

### 3.3. Aerogels as Innovative Sorbents

Silica aerogel (SA) is a suitable sorbent for use in industrial processes. It is a non-toxic, high-performance, and cost-effective component. This adsorbent is one of the porous forms of silica. It is the lightest synthetic solid. This material has mesopores with a large surface area, large pore volume, and high porosity. Due to their surface properties and good pore volume, they are used as sorbents for the purification of liquids and gases (suitable literature references can be found, for example, in the review [104]).

Hydrophobic SA is effective for the removal of both soluble and insoluble contaminants in water. They remove various pollutants such as paints, heavy metals, radioactive pollutants, pesticides and herbicides, phenolic compounds, pharmaceuticals, nitrates, etc. However, different pollutants require SA with specific properties. Chemically, SA is simple, but its production is a sophisticated procedure, taking place in the basic scheme of the sol/gel method in the following steps: (a) Gel preparation: The solution is prepared from a source solution of silica, and gelation occurs after the addition of a catalyst. Gels are usually divided according to the dispersion medium used, e.g., into hydrogel or aquagel, alcogel, and aerogel (for water, alcohol, and air, respectively). (b) Gel aging: The gel prepared in the first step is allowed to age in the mother liquor. This aging process solidifies the gel so that the shrinkage during the drying step is minimal. (c) Gel drying: In this step, the gel should be freed from pore liquid. To prevent the collapse of the gel structure, drying takes place under special conditions. The source substances-precursors are: tetramethoxysilane, tetramethoxysilane, methyltriethoxysilane, polyethoxydisiloxane, and water glass.

Each mix of precursors can strongly influence the texture and hydrophobicity of the final products. Various solvents are used to dissolve the precursors, e.g., alcohols, acetone, dioxane, tetrahydrofuran, and ammonium hydroxide. Different combinations of these components influence the hydrophobicity of the final sorbent and, thus, the preferred use for the sorption of various hydrophobic pollutants. Different catalysts are used. The sol/gel process of SA production seems simple, but all three mentioned stages influence the final properties of this sorbent significantly. For example, the final stage of gel drying is crucial. In the literature, one can find a number of experimental studies of the preparation of this aerogel, which is also applied for a number of other purposes, e.g., for thermal insulation. Their overview would represent separate research. Here, we only list the most important ones, such as [104,105,106] (Although older, it can be recommended, as the capacity measurements show that hydrophobic silica aerogels modified in this way are excellent adsorbents for various toxic organic compounds from water. Compared to granular activated carbon (GAC), they show capacities that are 15 to 400 times higher for all tested compounds). The adsorption properties of hydrophobic silica aerogel remained stable even after 20 adsorption/desorption cycles; toluene, benzene, ethylbenzene, chlorobenzene, chloroform, etc., were sorbed; and sorbents were regenerated by thermal desorption [107].

In laboratory sizes, the sorption of micropollutants on a large number of aerogels is tested. These are, for example, aerogels based on cellulose, graphene, or a joint composite [108]. The basic materials for the preparation of aerosols include carbon aerogels based on graphene and carbon nanotubes as sorbents. However, their preparation involves more laborious production processes, which require higher costs. However, there are also simple solutions, e.g., based on waste paper. It is an application of the sol–gel method, carried out by mixing paper in water with subsequent deep lyophilization when the solid lyophilizate exhibits a large interfacial area and high porosity. The products can be used as sorbents, e.g., for removing antibiotics from water [109]. Carbon aerogels based on graphene and carbon nanotubes as sorbents are the basic material for the preparation of aerosols; however, their preparation involves greater costs and more laborious production procedures. In general, biomaterials such as cellulose, starch, chitosan, chitin, carrageenan, and graphene have been intensively studied for the production of sustainable aerogels for water treatment. Cellulose, which is abundant in nature, has received considerable attention in recent years. Ahmad et al., 2023 [110] highlight the potential of cellulose-based aerogels as a sustainable and efficient material for the removal of dyes and heavy metals from water during the treatment process.

Aerogels based on biomaterials (e.g., cellulose and chitosan) are a simple solution. It is connected with the production of simple innovative sorbents, capable of sorbing both metals (improved, e.g., by modification with amino groups) and organic micropollutants. Relevant literature covering various modifications of such bioaerosols is in the original work, e.g., by [111]. Moreover, current research focuses on the synthesis of three-dimensional aerogels, e.g., based on Ti-MXene/graphene, using a simple hydrothermal method of self-assembly with subsequent freeze-drying [112]. The result of the integration of bimetallic MXene and graphene aerogel is an impressive broad-spectrum ability to remove multiple organic contaminants from wastewater. The method is simple for research purposes; nevertheless, it will probably not have a more realistic application in large volumes of sorbent preparation.

A demonstration of the preparation of a bio-aerogel with excellent sorption properties, which could be applied economically within the framework of the intentions of the circular bioeconomy, is based on microalgae. Microalgae can be cultivated on waste nutrients and in large bioreactors. Experience in their cultivation is wide, e.g., several decades of gaining experience in the Czech Republic. Microalgae, e.g., Chlorella, are separated by coagulation, brought into contact with FeCl_3_ (traces of chitan accelerate coagulation), and dried. The algal biomass is then mixed with an alkaline solution (pH 12) and sodium dodecyl sulfate (a common anionic surfactant), dried, collected by centrifugation, and deep-dried-lyophilized by freezing to form an aerogel [113]. A number of related aerogels have been mentioned in the scientific literature; however, only very few are available on the market [114].

Despite the promising applications of aerogels and their high potential to be used in pollutant removal, particular research is still needed to introduce these materials to the sorbent market. Within this context, it is crucial to consider that highly energy-intensive processes (i.e., freeze-drying or CO_2_ drying) are usually required to produce aerogels. Consequently, a techno-economic analysis, together with kinetic and gelation studies, can be useful to determine the correct reactor configurations and drying times depending on the material being produced. Furthermore, these types of analyses would enable the optimization of experimental conditions and help improve the aerogel preparation process in terms of economic viability, even for large water flows.

It is proposed that simple synthesis methods involving less complicated drying, freezing, and processing steps should be developed [115]. It would have less environmental impact and would be non-toxic. Such methods must be easily scalable from laboratory to full scale and commercial use. Since the regeneration of aerogel-based adsorbents often leads to secondary contamination, a number of studies should be carried out regarding downstream processing to ensure an effective strategy addressing the fundamental dimensions of environmental sustainability in removing harmful secondary products from water. This is generally true considering the sorption by different sorbents. In addition, a detailed techno-economic and life cycle feasibility analysis of aerogels should be carried out to ensure their real feasibility, including their use. Laboratory studies with in-depth analyses of the adsorption mechanisms are also needed to further evaluate the potential use of aerogel in the removal of emerging pollutants such as persistent organic pollutants (POPs) and perfluoroalkyl acids (PFAS).

Presently, there is an effort to modify aerogels with carbon structures [116,117], amines [103,118], or pure graphene aerosols as completely new sorbents [119] aimed at modifying the sorbent surface by introducing new functional groups and enriching the sorption capacities. These are good sorbents for laboratory verification and publications but apparently far from applications on industrial scales.

Here, we very modestly draw attention to the possible real applications of these types of innovative sorbents, especially biogels; nevertheless, presently, this is probably still a matter of a rather distant future; see, e.g., the considerations of [114].

## 4. Conclusions

Innovative sorbents are receiving increasing attention related to their excellent physicochemical properties. Carbon nanomaterials, specifically carbon nanotubes, fullerenes, graphene, graphene oxide, and activated carbon, have great potential for removing heavy metals from water due to their large surface area, nanoscale size, and availability of various functionalities; moreover, they can be more easily chemically modified and recycled. However, the transition from laboratory discoveries to scalable, cost-effective, and real-world applications faces obstacles, especially in adapting these innovations to practical use. Artificial intelligence and machine learning offer promising solutions, optimizing adsorption processes and performance of innovative nanomaterials in different environments, potentially bridging the gap between research and applications. However, we are still far from viable and economically sustainable sorption technologies. Future research needs to focus on efficient processes requiring only small concentrations of nanomaterials and on developing cost-effective methods for their synthesis and large-scale testing of their effectiveness for successful field applications. In conclusion, regarding sorption, activated carbon remains the most effective material for the removal of emerging pollutants and the main sorbent in wastewater treatment plants, together with biochar.

## Data Availability

Data are contained within the article.

## Abbreviations

The following abbreviations are used in this manuscript:

ampC	Antibiotic Resistance Gene of *β*-Lactam
BDC	1,4-benzenedicarboxylate
BDMHDA	benzyldimethylhexadecylammonium
CNTs	Carbon nanotubes
DDAB	Didodecyldimethylammonium bromide
DMF	dimethylformamide
ecfX	Antibiotic Resistance Gene
ermB	Antibiotic Resistance Gene of Macrolide
GO	Graphene oxide
HDTMA	hexadecyltrimethylammonium bromide
HPVP	poly-4-vinylpyridine-co-styrene
MOF	Metal organic framework
NZVI	Nano-zero valent iron
ODTMA	octadecyltrimethylammonium
PET	Polyethyleneglycol terephthalate
PFAS	polyfluoroalkyl substances
PFOA	perfluorooctanoic acid
POE	*Portulaca oleracea* L. extract
POPs	Persistent Organic Pollutants
rGO	Reduced graphene oxide
SA	Silica Aerogel
Sul2	Antibiotic Resistance Gene of Sulfanilamide
tetA	Antibiotic Resistance Gene of Tetracycline
WWTPs	Wastewater treatment plants
